# Innovative Photonic Sensors for Safety and Security, Part II: Aerospace and Submarine Applications

**DOI:** 10.3390/s23052417

**Published:** 2023-02-22

**Authors:** Antonello Cutolo, Romeo Bernini, Gaia Maria Berruti, Giovanni Breglio, Francesco Antonio Bruno, Salvatore Buontempo, Ester Catalano, Marco Consales, Agnese Coscetta, Andrea Cusano, Maria Alessandra Cutolo, Pasquale Di Palma, Flavio Esposito, Francesco Fienga, Michele Giordano, Antonio Iele, Agostino Iadicicco, Andrea Irace, Mohammed Janneh, Armando Laudati, Marco Leone, Luca Maresca, Vincenzo Romano Marrazzo, Aldo Minardo, Marco Pisco, Giuseppe Quero, Michele Riccio, Anubhav Srivastava, Patrizio Vaiano, Luigi Zeni, Stefania Campopiano

**Affiliations:** 1 Dipartimento di Ingegneria Elettrica e delle Tecnologie dell’Informazione, Università degli studi di Napoli Federico II, Via Claudio 21, 80125 Napoli, Italy; 2 Istituto per il Rilevamento Elettromagnetico dell’Ambiente, Consiglio Nazionale delle Ricerche, Via Diocleziano 328, 81024 Napoli, Italy; 3 Dipartimento di Ingegneria, Università degli Studi del Sannio, Corso Garibaldi, Palazzo Bosco Lucarelli, 82100 Benevento, Italy; 4 European Organization for Nuclear Research (CERN), CH-1211 Geneva, Switzerland; 5 National Institute for Nuclear Physics (INFN), 80125 Napoli, Italy; 6 Dipartimento di Ingegneria, Università della Campania Luigi Vanvitelli, Via Roma 29, 81031 Aversa, Italy; 7 Optosensing Ltd., Via Carlo de Marco 69, 80137 Napoli, Italy; 8 Dipartimento di Ingegneria, Università degli studi di Napoli Parthenope, Centro Direzionale Isola C4, 80143 Napoli, Italy; 9 Istituto per i Polimeri, Compositi e Biomateriali Consiglio Nazionale delle Ricerche Via Enrico Fermi 1, 80055 Portici, Italy; 10 CERICT SCARL, CNOS Center, Viale Traiano, Palazzo ex Poste, 82100 Benevento, Italy; 11 Optosmart Ltd., Via Pontano 61, 80122 Napoli, Italy

**Keywords:** optical fiber sensors, fiber bragg gratings, distributed sensing, aerospace structure monitoring, submarine monitoring

## Abstract

The employability of photonics technology in the modern era’s highly demanding and sophisticated domain of aerospace and submarines has been an appealing challenge for the scientific communities. In this paper, we review our main results achieved so far on the use of optical fiber sensors for safety and security in innovative aerospace and submarine applications. In particular, recent results of in-field applications of optical fiber sensors in aircraft monitoring, from a weight and balance analysis to vehicle Structural Health Monitoring (SHM) and Landing Gear (LG) monitoring, are presented and discussed. Moreover, underwater fiber-optic hydrophones are presented from the design to marine application.

## 1. Introduction

Photonic sensing technologies and optical fiber sensors are increasingly being used in new and emerging sectors for a variety of measurement and monitoring applications. Our multidisciplinary group has been active in this field for the last twenty years, concentrating most of our attention on the usage of photonics and nanophotonics [1,2,3,4,5,6,7,8,9,10] for both medical and industrial applications [11,12,13,14,15,16,17,18,19,20], by which we have realized a large variety of in-field applications [21,22,23,24,25,26,27,28,29,30,31,32], some of which have reached the market or are operative in some industrial or research plants (e.g., CERN, Geneve, railways, and aerospace) [33,34,35,36,37,38,39,40,41,42,43,44,45,46,47,48]. Hence, we have decided to collect, in this and its two companion papers [49,50], our main results concerning both safety and security in the environment. This paper is the second one (Part II) and focuses on the applications, whereas fundamental concepts of the used technologies are discussed in [49].

This manuscript focuses on innovative photonic sensor applications in the aerospace and submarine context, according to which this paper is ideally divided into two parts. First, we analyze the problem of the aircraft weight and balance, describing a new approach to finding the center of mass of an airplane before takeoff; then, some innovative solutions for Structural Health Monitoring (SHM) and Landing Gear (LG) monitoring are described. In the second part, we focus our attention on the submarine environment with particular attention to a new class of underwater fiber-optic hydrophones to be used as sonar in towed array applications.

## 2. Aircraft Monitoring

### 2.1. Design and Characterization of a Sensorized Carbon Fiber Panel for Anti-Ice Applications in Aeronautical Field

Due to its characteristics widely discussed in this paper, fiber-optic sensors are arguably the most suitable solution for harsh environments in which temperature or strain monitoring is needed. Particularly, since naked fiber Bragg grating (FBG) sensors are very thin (hundreds of µm) and light, it is easy to embed them in many materials in a non-invasive way without altering the structure. Moreover, FBG sensors can be effortlessly used to measure temperature values around 0 °C, monitoring the presence of ice.

As a matter of fact, the presence of ice can be very dangerous in some circumstances, such as during the flight of an aircraft at a high altitude. During a normal flight, the boundary conditions change from a temperature value of −40 °C with 0% of humidity to high relative humidity (if moisture is present), provoking icing conditions due to the wet air and the extremely cold surface of the aircraft. Issues occur if the ice grows on the wings leading edge, causing loss of altitude and engine problems [51]. For the mentioned reasons, at the state of the art, many companies and scientists are more and more focusing on the research of a system capable of avoiding these dangerous conditions, removing the ice in a proactive way. Many publications were completed by employing the air flux generated by the engine [52]. Other solutions regard nanostructured surfaces [53], airfoil modeling [54], and short pulse laser surface [55], seemingly far from a consolidated solution.

In the proposed application, an anti-ice system based on composite materials was designed and tested. The composite material employed is carbon fiber, in which several conductive wires were inserted during the fabrication process. The wires are externally insulated, avoiding unwanted short circuits. The FBG structure, already used in some studies related to carbon fiber [56], in this study behaves as a temperature monitoring sensor, indicating whether the temperature is high enough (no ice is present) or if the temperature is decreasing excessively (presence of ice growing). After a preliminary study, followed by a numerical simulation to understand the feasibility of the system, a test structure was fabricated and tested at room temperature, validating the usage of FBG sensors with a thermal imager.

#### 2.1.1. Problem Definition and Measurement Setup

**Figure 1:**
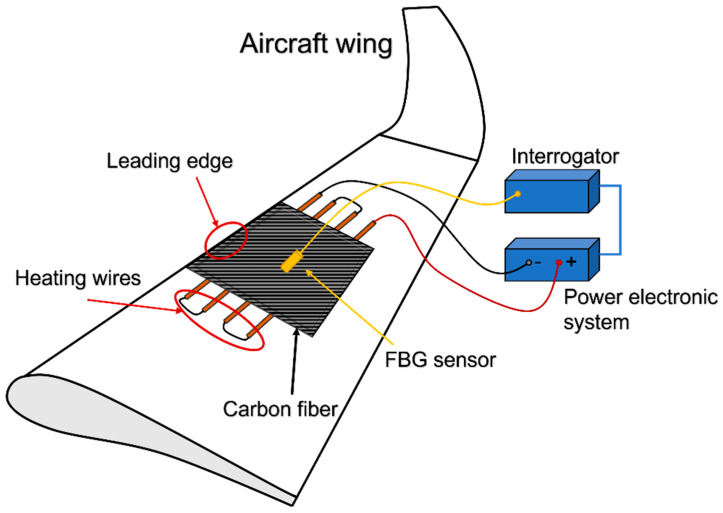
Block scheme representing the proposed solution.

To overcome the mentioned safety problem in the aeronautical environment, the anti-ice system described was designed. The comprehensive system can be described in three main parts (shown in Figure 1):

- The sensor part (yellow) is composed of one or more FBG sensors;
- The heat part (orange wires inside carbon fiber) is composed of a set of conductive wires working as heat source due to Joule effect;
- The acquisition part (blue blocks) is composed of the FBG interrogation system and a power electronic system, which activates the heat source if a temperature threshold is overcome.

Hence, the system working principle can be described as follows: the FBG sensors, which can be installed both inside and on the top/bottom surface of the carbon fiber, register the in-time weather conditions in the points they are located; signals back-reflected from them are acquired by a control unit. The latter, after signal processing, allows controlling the flowing current into the conductive wires that, through the Joule effect, boost the temperature up to the carbon fiber surface, mitigating or avoiding ice formation. Moreover, the thermal control inducted by the FBG sensors is also employed as feedback for the flowing current, interrupting it if the boundary conditions are such that the heat source is not needed anymore.

**Figure 2:**
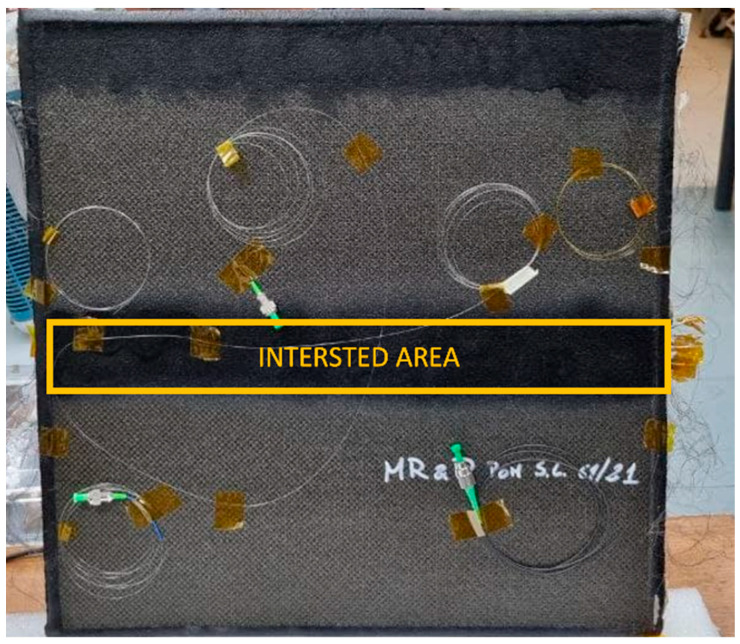
Picture of the test piece, highlighting the interested area.

To prove the feasibility of the system, the test-structure was then fabricated according to the numerical results. It was composed of 10 layers of carbon fiber material (each layer of 0.208 mm height) in an area of 40 cm × 40 cm with 160 wires characterized by a diameter of 0.1 mm (Figure 2). A test was conducted also embedding a stretch of fiber optic within the composite material, evaluating the final structure with non-destructive analyses. The results led to understanding that the presence of the fiber optic does not affect the structure of the composite material and that it is possible to embed a FBG sensor between carbon fiber layers.

**Figure 3:**
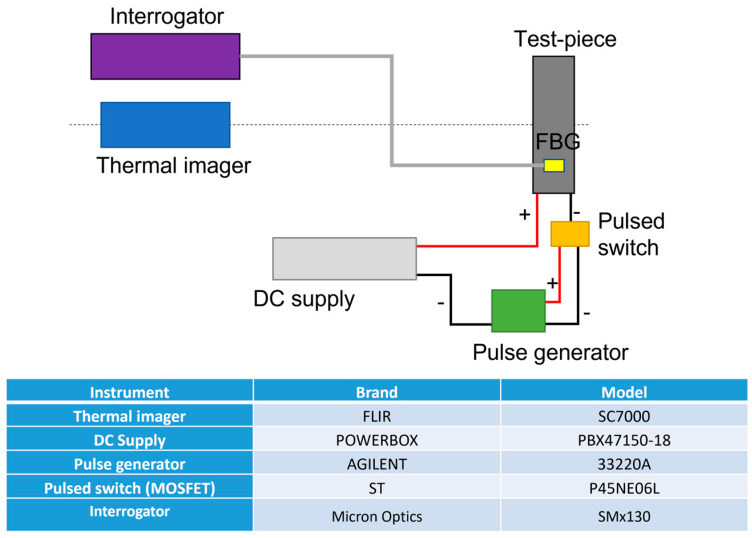
Block scheme regarding the measurement setup with (in table) info on the instrumentation employed.

**Figure 4:**
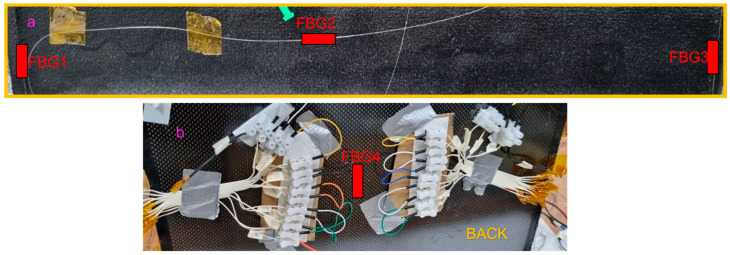
Representation of FBG sensor positioning during the measurements.

Tests at room temperature were conducted. The task was to increase the temperature on the carbon fiber surface until reaching a deviation of 10 °C. Due to the significant number of heating wires, an area of 5 cm × 40 cm was considered, connecting 14 wires. The block chain representing the setup is shown in Figure 3. It is worth noting the presence of the pulsed switch. This was included to modulate the current flowing through the heat source, modulating the duty cycle of the square wave driving the gate of a MOSFET with the drain connected to the negative pole of the heating wires and the source at the ground. The heat source, composed of 14 wires, was directly connected with the positive pole to the DC supply. The temperature measurement relied on two different technologies:

▪ Four FBG sensors (shown in Figure 4), where two are located at the edge, one in the middle part (naked FBGs), and the last one on the back (ceramic packaged FBG). The sensors were interrogated with a Micron Optics smx130;
▪ A thermal imager focused on the tested area.

The thermal imager was employed to register a thermal mapping on the whole area under the test and check if unwanted hot-spot phenomena were present due to possible short circuits among the wires. Furthermore, the thermal imager was used to validate the temperature measured by FBG sensors.

#### 2.1.2. Results

As the first analysis, the network composed of 14 heating wires was characterized electrically. From a measurement between the 1st and the 14th, a high resistance value (over 3 MΩ) was present, highlighting overall isolation among the connected wires. Furthermore, the resistance value among adjacent wires was measured as well, also obtaining, in this case, values over hundreds of kΩ. The equivalent resistance, obtained from the wires’ series connection was 470 Ω, covering an area of 5 cm × 40 cm. Considering the nominal wire resistance of about 69 Ω/m and a length of about 50 cm, the equivalent resistance value implies a mean value of about 33 Ω per wire, in line with the nominal value.

**Figure 5:**
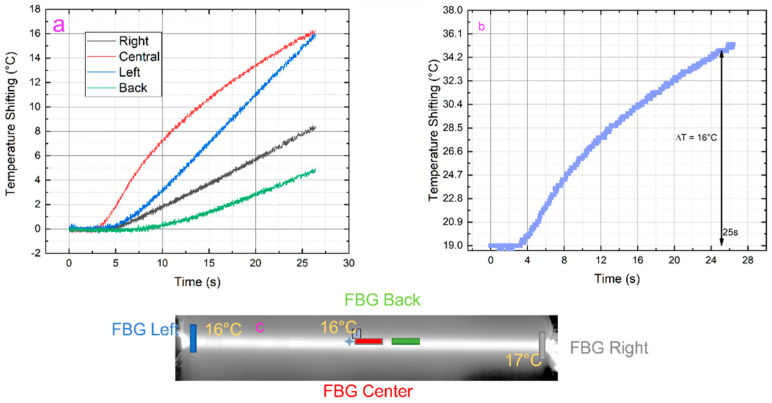
Temperature measurement through 4 FBG sensors (**a**); same temperature obtained through thermal imager after 25 s in the middle of the interested area (**b**); heat mapping of the thermal imager temperature measurement with highlighted temperature deviation at left, right, and center of the interested area (**c**).

In Figure 5, the results are shown. The heat source was ignited with 100 V for a total power of 21 W (1 kW/m^2^). In Figure 5c, it can be noted the thermal mapping acquired by the thermal imager on which three points of measurement are highlighted: left, right, and center. The temperature reported in each point (left, right, and center) is referred to the deviation compared to the room temperature of 19 °C (initial temperature shown in Figure 5b), calculated as the mean value on the region of interest. The thermal imager acquisition set was 25 s, as per the constraints imposed by the aircraft company. A very interesting result is shown in Figure 5a,b, reporting the temperature measured from 4 FBGS (a) and the temperature trend at the center of the interested area. The two curves follow the same trend and final value, while both right and left sensors reported the same value compared to the one processed by the thermal imager. Regarding the FBG placed on the back, it exhibits a slow dynamic since the FBG was surrounded by a ceramic package, spending more time to reach the steady state than the FBG naked one.

In conclusion, this system takes advantage of the FBG peculiarities to mitigate the anti-ice problem during the flight. From the reported analyses, the system can heat the carbon fiber surface to 16 °C in 25 s with 1 kW/m^2^ of power. Other tests in the climate chamber will follow.

### 2.2. Aircraft Weight and Balance

Since 2015, our research group has started a feasibility study for a novel weight and balance (W&B) measurement system in aircraft landing gears based entirely on FBG sensor technology [57].

The LG is among the major elements of an aircraft whose main aim is to furnish a suspension system in the course of taxing, takeoff, and landing. The LG is conceived to be able to absorb and dissipate the kinetic energy arising from the impact, thus mitigating the related forces transferred to the airframe. It also facilitates its deceleration by a wheel braking system and enables its directional control on the ground by a wheel control system [58,59,60,61,62,63,64,65,66].

**Figure 6:**
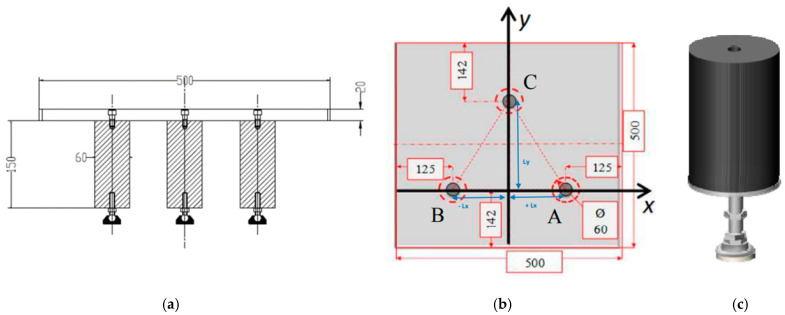
(**a**) Schematic representation of the mock-up structure; (**b**) drawing of the metal plate; and (**c**) pvc support. Adapted from Ref. [57].

In this context, we have presented the successful use of an FGB sensor system for continuous and remote measurement of the load acting on real aircraft LGs [66,67]. The basic idea is based on the determination of the instantaneous values of the weight and the coordinates of the center of gravity (CoG) of an aircraft by measuring the loads acting on the main and nose LGs using an appropriate combination of strain data provided by the FBG sensor network. To validate the proposed system, we designed, conceived, and developed a laboratory demonstrator (see Figure 6) consisting of an adjustable tripod stand made of an aluminium plate (simulating the aircraft fuselage) and supported by three polyvinyl chloride cylinders (mimicking the three LGs), each one equipped with an FBG strain sensor. Cylinder “C” simulates the nose LG, whereas cylinders “A” and “B” simulate the main LGs.

With reference to Figure 6b, we know that simply placing a mass M on the aluminum plate and changing its position (x, y) results in a change in the location of the barycenter of the demonstrator. The deformation caused by the placement/movement of the mass M on each PVC support can be monitored by a suitable strain sensor system that allows the force acting on each support, and thus the weight of M, to be determined. Given the geometric configuration of the three supports and the force acting on each of them, we can also derive the CoG. As reliable strain sensors to accurately determine the deformations of the three supports, we used fully polyimide-coated FBG (5 mm long) suitably bonded to the side surfaces of each PVC support.

**Figure 7:**
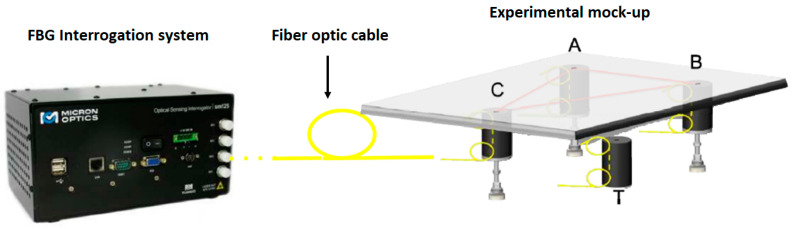
Experimental setup. Adapted from Ref. [57].

A comprehensive experimental analysis was performed by applying known weights to the plate placed at specific coordinates and then retrieving all values of weight and CoG from the strain data provided by the FBGs after eliminating temperature effects. To this aim, another FBG was bonded to the side surface of an additional PVC cylinder (referred to as “T” in Figure 7). The “T” cylinder is similar to cylinders A, B, and C but is not screwed to the metal plate in such a way that it responds only to temperature variations.

To compare the results of the experimental analysis with the theoretical ones, we also developed a suitable numerical model of the laboratory demonstrator. It allows the accurate determination of the weight and CoG position of the demonstrator system based on the forces acting on each cylinder due to the application/movement of the mass M on the plate.

**Figure 8:**
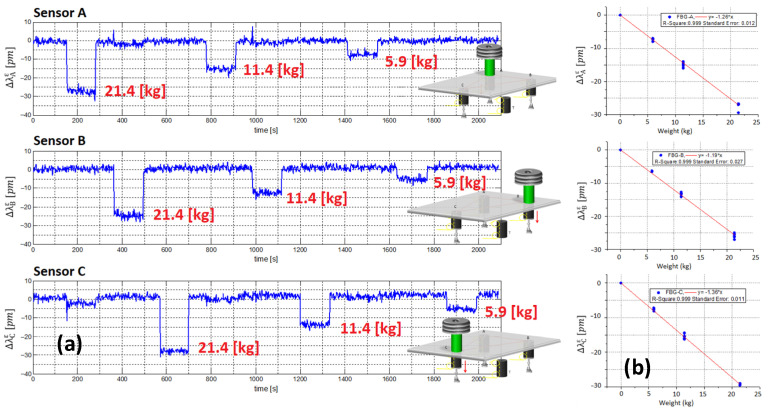
(**a**) FBG sensor responses during the calibration tests and (**b**) calibration curves of the three FBG strain. Adapted from Ref. [57].

Before performing the experiments, we carried out a calibration (in terms of $\Delta \lambda_{i}^{\varepsilon }-P_{M}$, where $\Delta \lambda_{i}^{\epsilon }-P_{M}$ is the induced wavelength shift, and $P_{M}$ is the weight related to the mass M) of the FBG strain sensors glued to the three mounts by placing known weights (21.4, 11.4, and 5.9 kg) on the metal plate in correspondence with each cylinder. Figure 8a shows the wavelength shift of the FBG sensors during the calibration tests carried out with the known weights located in three different positions (position A, B, and C). As expected, the $\Delta \lambda_{i}^{\varepsilon }$ provided by each strain sensor is zero when the mass is applied in correspondence with the other cylinders and increases with the weight of M when it is applied in correspondence with the cylinder to which they are bonded. Figure 8b shows the calibration curves of the three strain sensors, all of which exhibit linear behavior. Considering the slope of the linear fitting curves, the sensitivity of each sensor can be determined as a function of the applied load (they are reported in the insets of Figure 8b).

After completing the calibration test, we conducted an in-depth experimental campaign by applying a known weight to the plate (at different positions along the x- and y-axes) and determining the applied weight and the CoG coordinates of the demonstrator based on the responses of the FBG strain sensors and the theoretical model.

**Figure 9:**
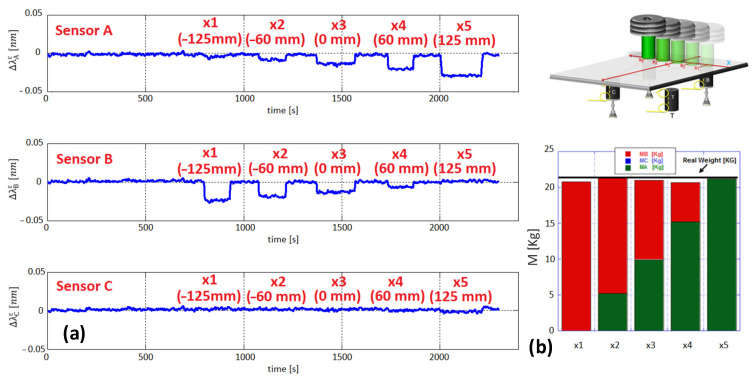
(**a**) FBG sensor responses during the tests along x-axis and (**b**) estimated weights. Adapted from Ref. [57].

Figure 9a shows the wavelength shift of the FBG strain sensors when the mass M (weighing 21.4 kg) is moved along the x-axis (y = 0 and x = x_1_, x_2_, x_3_, x_4_, x_5_), from position x_1_ (−125 mm, i.e., point B) to position x_5_ (125 mm, i.e., point A). As theoretically expected, $\Delta \lambda_{c}^{\varepsilon }$ is zero since all forces act only on cylinders A and B (located along the x-axis).

Based on the $\Delta \lambda_{i}^{\varepsilon }$ returned by each FBG strain sensor and using the sensitivity coefficient $\alpha_{i}$ determined during the calibration tests, the total applied weight can be reconstructed by summing the individual force contributions observed on each support. The weights for all x-positions in the histogram of Figure 9b show very good agreement between estimated and actual values.

The weight values for all x-positions, calculated by the method described above, are shown in the histogram of Figure 9b. The green and red columns show very good agreement between estimated and actual weight values (black line). The obtained results agree well with those calculated using a suitably developed mathematical model and reveal that the proposed W&B architecture is capable of determining the weight applied to the laboratory demonstrator with an accuracy < 2%, as well as the CoG coordinates with millimetric precision.

In 2018, we presented results on the use of a FBG sensor network integrated on an aircraft LG system for remote and real-time load determination. FBG strain sensors were integrated in different locations of real LGs, both on the main (MLG) and on the nose (NLG), provided by Magnaghi Aeronautica Spa [68].

FBGs (manufactured by HBM) were integrated into a very compact plastic housing able to confer the robustness and reliability required for a hard application such as the real-time load measurements on an LG structure. We identified two typologies of fiber-optic sensors, a monoaxial (“linear”) and a triaxial type, both of which can be integrated on flat and rounded metal components.

**Figure 10:**
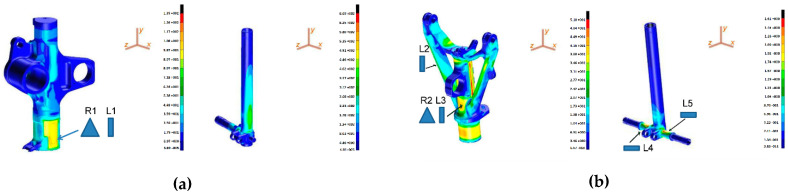
Map of the stress in static arrangement for (**a**) MLG and (**b**) NLG. (Reprinted/adapted with permission from Ref. [68]).

To help identify the best locations for the FBGs on the LGs, a static stress analysis of the two gears was performed using the finite element method (FEM) (the results are shown in Figure 10), which provides the stress distribution through the LG structures when subjected to the maximum load condition at the takeoff (approximately 4600 kg of applied load).

**Figure 11:**
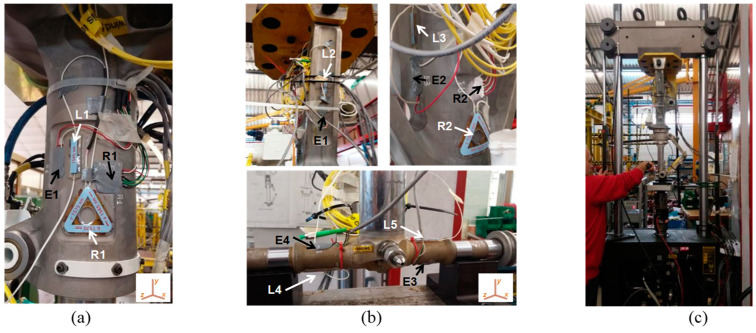
FBGs (white) and conventional strain gauges (black) integrated on the (**a**) main and (**b**) nose landing gear; (**c**) pictures of the experimental setup. (Reprinted/adapted with permission from Ref. [68]).

Suitable installation locations must exhibit significant deformations (i.e., significant sensitivity to the applied load) and geometrical features that allow proper installation of the sensors. With reference to Figure 11, we designed and implemented:

- for MLG, a fiber-optic sensor network composed of two FBGs, a uniaxial one (denoted by “L”) and a triaxial one (denoted by “R”), to install on the main fitting (L1 and R1);
- for NLG, a fiber-optic network composed of five FBGs, four in uniaxial configuration and one in triaxial: two monoaxial sensors to install in two opposite areas of the wheel axle (L4, L5) and the other FBGs to integrate in the main fitting (L2, L3, and R2).

In addition, several conventional (electrical) strain gauges were installed as reference for the strain data very close to their fiber-optic equivalent (Figure 11a,b).

**Figure 12:**
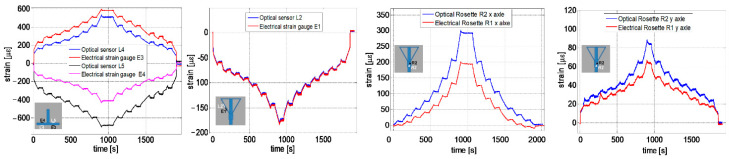
Optical and electrical strain gauges sensorgrams installed on the Nose Landing gear. Reproduced with permission from ref. [68] (the figure is released under a Copyright Clearance Center’s RightsLink^®^ service).

**Figure 13:**
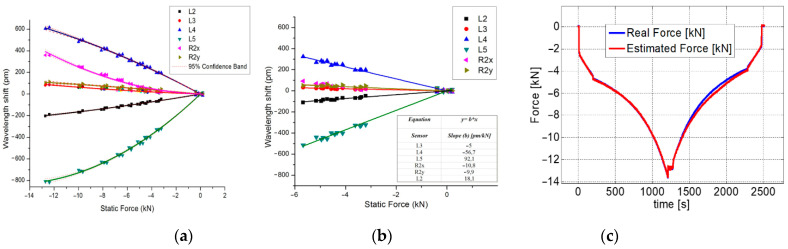
(**a**) Calibration curves of the sensors integrated on the NLG, as determined by 2 identical (step-by-step) experiments; (**b**) enlargement in the interval 0–6 kN; and (**c**) True force vs. Calculated one during the validating test performed using the NLG. Reproduced with permission from ref. [68] (the figure is released under a Copyright Clearance Center’s RightsLink^®^ service).

An extensive test campaign was conducted to qualify the proposed system by mounting the two LGs under a 25 kN hydraulic press connected to a PC, which enabled the exertion of a predefined and known load in the range of 0–25 kN, resulting in a variation in the LG shock absorber stroke (Figure 11c). In the first test, we verified the ability of each FBG to monitor the changes over time in the load exerted on the gear. The tests were conducted at room temperature with force applied to the LGs under the test gradually (stepwise) increased to achieve a change in the shock absorber stroke from 0 mm to 200 mm (max. takeoff weight, ~4600 kg) with increments of 25 mm per minute. Data provided by FBG sensors were compared with the data provided by the conventional strain gauges, and the results obtained for the NLG gear are shown in Figure 12. Similar results were obtained for the experiments on the MLG, and the sensorgrams clearly demonstrate the ability of the FBGs to accurately track the temporal changes in the exerted load for any step, as well as their worthy agreement with the reference devices. In addition, a test was performed to calibrate the FBG strain sensors and estimate the load. Figure 13a,b show the Δλ_B_ (nm) vs. the static force F (kN) exerted on the gear of the FBGs installed on the NLG, obtained from two repeated tests such as the one described above.

The calibration curves show that all FBGs exhibit a nonlinear Δλ_B_-F relationship throughout the force range (0–13 kN) regardless of their position on the gear. However, when the calibration curves are enlarged in the 0–6 kN interval, linear behavior can be noticed for each FBG. As expected, the graphs shown in Figure 13a prove that the sensors positioned on the wheel axle have the highest sensitivity to the applied load with respect to all other sensors (about 92 pm/kN).

After calibration, we performed another experiment to verify that the data provided by the FBG could be used to precisely calculate the load exerted on the NLG. To this aim, the stroke of the nose LG shock absorber was pushed at a rate of 10 mm/min, considering only one baseline at 200 mm, which corresponds to the highest weight at the takeoff. The retrieved results are shown in Figure 13c, where the exerted load, obtained by performing the average among the data returned by two fiber-optic sensors (L2 and L4), was compared with the actual load. The experimental results agree well with those obtained from conventional strain gauges positioned in proximity to their optical equivalents and provide evidence of the good potential of fiber Bragg gratings for remote and real-time load measurement on aircraft LG.

### 2.3. Structural Health Monitoring

In situ monitoring of aircraft structures ensure integrity with minimal need for human intervention. The development of “smart structures” goes beyond the concept of non-destructive inspection, as it relies on the use of in situ sensing to allow for rapid, remote, and real-time condition assessments. Optical fiber [69] distributed optical fiber sensors based on Rayleigh or Brillouin scattering are especially suitable, as they use the whole fiber as a sensor with a spatial resolution in the centimeter or lower than centimeter range.

In the following, we present two applications of distributed sensing for the aerospace industry, the former one based on a commercial interrogator based on Rayleigh scattering (Luna OBR 4600) and the latter one based on a custom interrogator exploiting Brillouin scattering.

**Figure 14:**
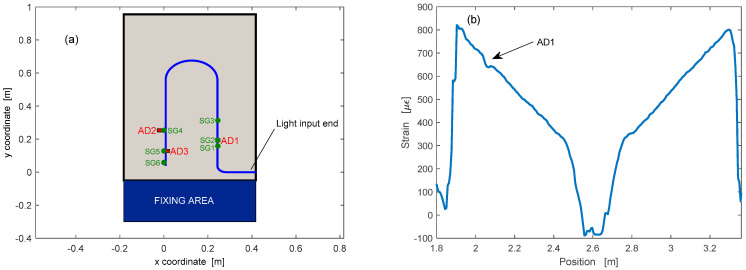
(**a**) Path of the optical fiber employed during the ground test (blue line), electrical strain gauges (green circles) and artificial delaminations (red patches); and (**b**) Strain profiles acquired by the optical fiber sensor for an applied load of 348 N.

For Rayleigh-scattering-based measurements, a composite panel of dimensions 1300 mm × 600 mm and a thickness of 3 mm constituted by 24 plies at 0 °C was chosen for our tests [70]. A standard single-mode optical fiber was embedded between the second and the third ply of the panel during fabrication, according to the scheme shown in Figure 14. The same figure also indicates the position of three artificial delaminations (AD), introduced into the panel by placing three 2 cm × 2 cm pieces of thin Teflon film within the layer occupied by the optical fiber. The artificial delaminations were added to verify the ability of the fiber-optic sensor to identify the presence of these defects through anomalies in the acquired strain distribution. Fiber-optic measurements were performed by connecting the fiber to the OBR 4600 reading unit and acquiring a reference trace under zero load conditions. This reference trace was then employed to determine the strain distribution resulting from the application of some load to one boundary of the panel while keeping fixed the opposite boundary. The strain distribution acquired with an applied load of 348 N is shown in Figure 14. The strain profile exhibits a linearly decreasing section corresponding to the rightmost fiber segment in Figure 14a, followed by a linearly increasing section corresponding to the leftmost fiber segment. These two linear sections are separated by a low-strain region corresponding to the bent part of the fiber path. The figure also highlights the local depression of the strain, occurring in correspondence with the Teflon patch AD1. This strain anomaly is due to the fact that the Teflon film reduces the adherence between the fiber and the composite plies, hence lowering the strain transfer efficiency.

**Figure 15:**
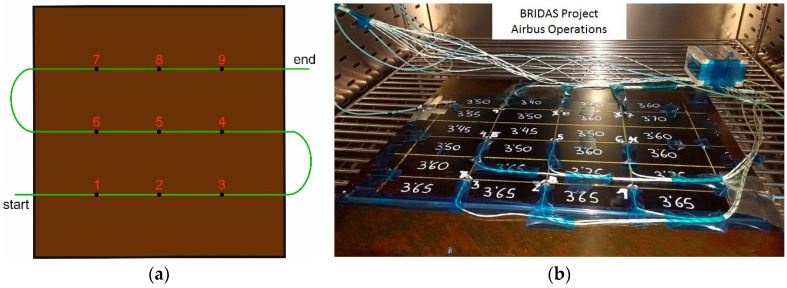
(**a**) Schematic view of the composite panel and optical fiber deployment; and (**b**) picture of the composite panel used for the tests.

Compared to Rayleigh-scattering-based sensors, those based on the Brillouin scattering have the advantage of being capable of absolute (not referenced) measurements. Therefore, they are capable of providing the absolute temperature using a previously calibrated optical fiber. In the reported experiments, a custom prototype based on the Brillouin Optical Frequency-Domain Analysis (BOFDA) [71] has been used to measure the temperature along an optical fiber attached over the surface of a composite panel (500 mm × 500 mm, 4 mm thickness) subject to temperature variations [72,73]. A schematic view and a picture of the panel are shown in Figure 15.

Furthermore, 8 thermocouples (TCs) were placed in positions #2 to #9 for temperature reference purposes. In Figure 15a, the green line indicates the path of the optical fiber used for BOFDA measurements, as well as that of a parallel fiber incorporating nine FBGs at the indicated positions.

**Figure 16:**
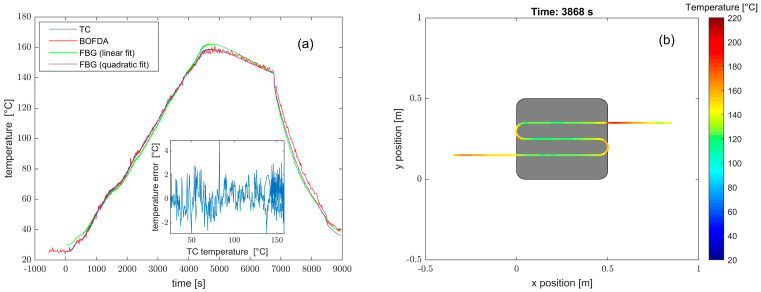
(**a**) Temperature evolution as acquired by the TC, the BOFDA sensor, and the FBG in the same position of the plate; and (**b**) temperature distribution as acquired by the BOFDA sensor in one instant of the heating phase.

Temperature cycles from room temperature to 200 °C were carried out by placing the panel in a furnace. We show in Figure 16a the temperature measured by the optical fiber at position #5, compared to those provided by the TC and the FBG at the same position. The inset shows the temperature deviation between the optical fiber sensor and the TC, characterized by an average value of 0.2 °C and a standard deviation of 1.02 °C. As a demonstration of distributed measurement, Figure 16b shows the temperature distribution along the fiber during the heating phase. As expected, during this phase, the fiber parts not in contact with the plate had a higher temperature than the plate-bonded fiber parts due to the thermal inertia of the plate.

### 2.4. Landing Gear Monitoring

Since the early 1980s [74], composite materials have started to be used in the manufacturing of aircraft components because of their lightness and their corrosion resistance to overcome the limits of aluminum and metallic items. Proper systems are needed to monitor the health state of the components made using these new materials [75]. Currently, the most widely used SHM technology for composite materials is based on optical sensors [76,77,78,79,80,81,82,83] due to their advantageous peculiarities compared to the traditional electrical strain gauges demonstrated over the past 20 years [84].

The sensors can be integrated on the composite structure to be monitored mainly in three different ways [85]:

- Bonding the FBGs on the surface of the composite structure with appropriate glues and procedures [86,87];
- Inserting the FBGs between two composite structures connected to each other in case of assembly of different parts;
- Incorporating the FBGs in the composite material during its production in order to protect the sensing devices from incidental contacts [75] and record damage inside the objects in multiple points [88].

The successful use of an FBG network for strain monitoring in aircraft LG components made in innovative composite materials was demonstrated in [89] within the Italian project “Carrello per Atterraggio con Attuazione Intelligente” (abbreviation CAPRI) (PON03PE 00135). In that work, the structural element called drag brace lower arm belonging to the nose landing gear adopted in the Alenia C27J aircraft (selected as the target sample to be made in composite materials) was sensorized and tested with increasing tensile loads.

Indeed, in the landing gear, the drag brace is one of the main load-carrying elements, which is loaded both in tension and compression [90] during takeoff and landing operations. The use of a composite material should target to both weight reduction and stiffness increase. However, their poor transverse and shear properties [91] introduce concerns about the impact behavior of the component. To improve the safety and keep the mettle of the component, an SHM is needed. FBG strain gauges are ideal candidates for the structural health monitoring of composite components since, as already said, they should be embedded within the structure without threatening its strength and stiffness [92]. Moreover, their multiplexing feature enables the mapping of the component deformation. Below, it is shown how the FBGs were applied successfully as strain sensors and as an alternative to accelerometers for the assessment of structural damages [93].

**Figure 17:**
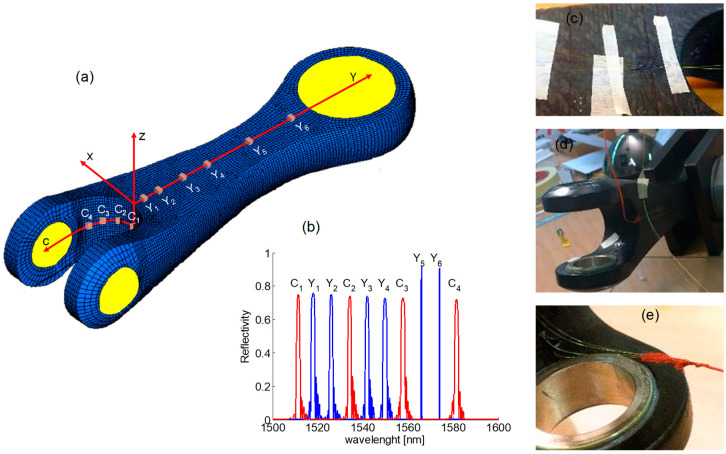
(**a**) Scheme of the arrangement of FBG sensors along Y-array and C-array, (**b**) spectra of the FBGs in the two arrays, (**c**–**e**) photos of the real item with the sensors (Adapted from Ref. [89]).

From a numerical analysis of the 3D model of the item under test, two custom arrays were designed to record the strain in the longitudinal direction and the strain in the bent region [89]. The first array was located in the central part of the lateral surface along the y-axis and included six FBGs, and the second array included four FBG sensors and was placed in the bent region to be monitored along the bent c-axis, as Figure 17 shows. All FBG sensors were bonded on the item by the cyanoacrylate adhesives Loctite Super Attak.

The sensorized item was subjected to three traction tests with different maximum loads, and the behavior of the sensor arrays was compared and analyzed.

**Figure 18:**
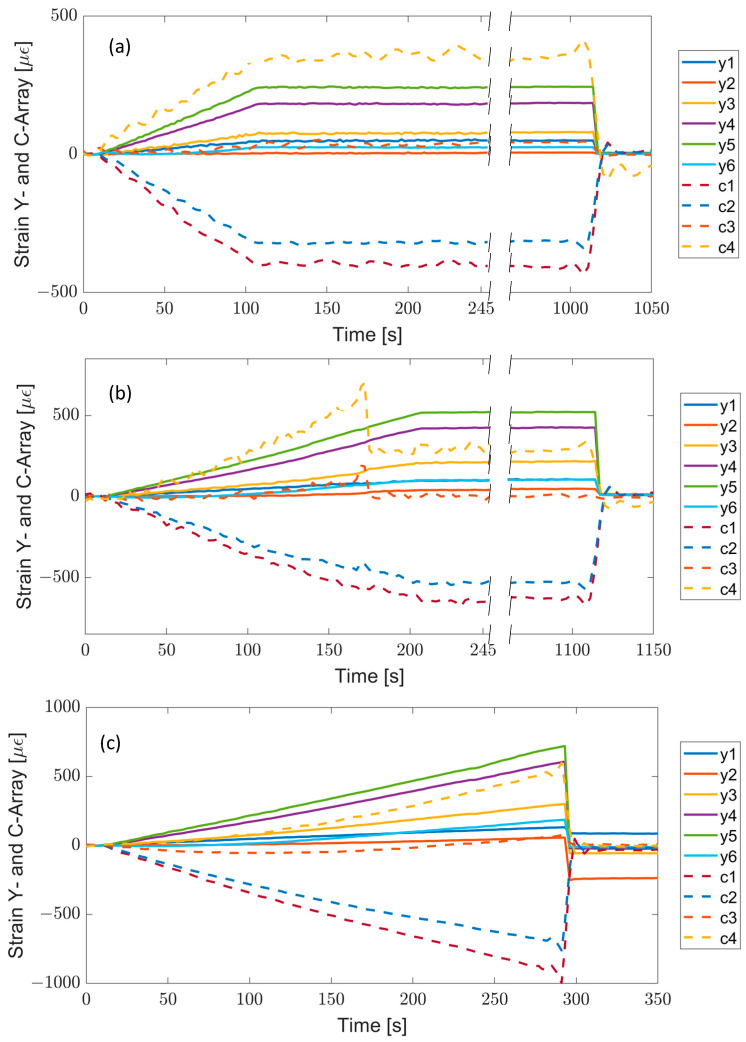
Time response of all FBG sensors in different test sections: (**a**) test A, (**b**) test B, and (**c**) test C. (Adapted from Ref. [89]).

Figure 18 reports the temporal evolution of the strains measured by the two arrays during the three tests: (i) in the first case (Figure 18a), the tensile load increases linearly from 0 to 25 kN in the time interval 10 ÷ 110 s, (ii) in the second experiment (Figure 18b), the maximum load is 50 kN (at 210 s), and, finally, (iii) in the third test (Figure 18c), the maximum load is 75 kN.

As expected, the FBGs of the Y-array always recorded positive strains, which increase moving from Y1 to Y5 because of the reduction in the cross-section area of the item under test, in accordance with the numerical simulations. The sensor Y6 measures strain values much lower than Y5 due to the larger cross section and the closeness to the metallic graft in the upper part of the item (fixing point).

A different speech must be made for the C-array, which monitors a region with more complex geometry.

The FBG C1, bonded in the central region between the two metallic ferules B1 and B2, because of its location, records the strain profile along the x-axis and reads a negative strain (compression) during the traction tests. As you move along the C-array, the direction of the strain sensors changes from the x- to the y-axis, then the effect of the compression along the x-axis decreases, and an elongation along the longitudinal direction y-axis appears. Consequently, C4 measures the highest positive strain.

Regarding the first test, the sensors show a linear response with the applied load with good stability and reversibility. The same behavior is observed in the second test until instant 170 s, when an irregularity in the sensors’ response of the C-array happen. That sudden response change indicates a break in some composite layers of the item (invisible from the outside). However, the y-array sensors continued to measure increasing strains until the load reached the maximum value of 50 kN. In addition, all the sensors kept their responses constant during the load holding and returned to their initial values when the load was removed.

Finally, in the last test, the maximum set value of 75 kN for the load was not reached because the composite item broke when the applied load reached 70 kN. After this event, all sensors recorded strains close to zero (initial value). Only sensors Y1 and Y2, because of their proximity to the break region, did not recover perfectly.

**Figure 19:**
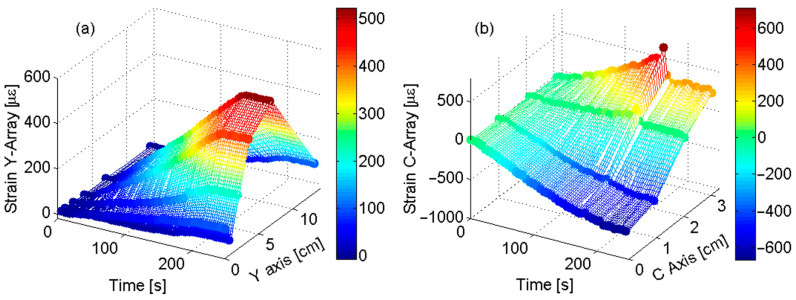
FBG Surface strain profile vs. time: (**a**) strain profile along y-axis; and (**b**) strain profile along c-axis (Adapted from Ref. [89]).

In conclusion, Figure 19a,b report 3D real-time maps for the y-axis and c-axis, respectively, giving a clear understanding of the surface changes in the sensors’ regions. In Figure 19b, it is clearly identifiable the unexpected situation at 170 s demonstrates the ability of the system to detect material failures early.

The tests in this work showed the usability of the FBG-based technology in aeronautic applications for the real-time monitoring of the structural health state of composite items.

**Figure 20:**
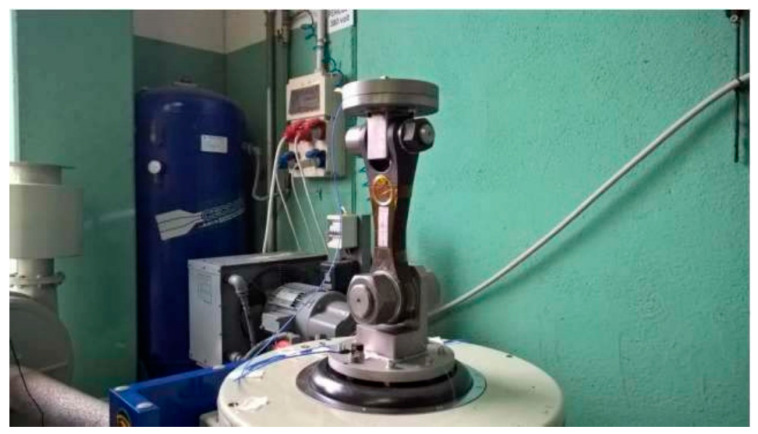
Experimental test setup for the vibration tests.

Moreover, to deeper investigate the composite landing gear reliability, vibration tests [94] were performed on the drag link component. To replicate the real operative condition for the system, one side is bolded on a flange fixed on the vibrating plate while the other side is pinned with a weight reproducing the landing gear components, as shown in Figure 20, where the single shaker system is visible.

**Figure 21:**
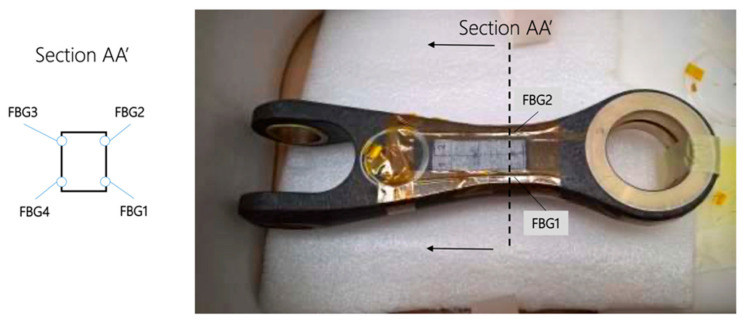
FBG installation of composite drag link.

Vibration characteristics of the components have been recorded by 2 triaxial accelerometers, which have an average sensitivity of 0.06 mV/g and a range of ±10 g. To measure the component response to the dynamic excitation, four FBG sensors were installed on the components, as shown in Figure 21. The vibration tests, performed according to the MIL-STD-810G-514.6 procedure, consisted of random vibrations and swept-sine solicitations at a sampling rate of 2500 Hz in the frequency range of 5–1000 Hz. Frequency response functions are computed to determine the resonant frequencies and the associated mode shapes and damping coefficients.

**Figure 22:**
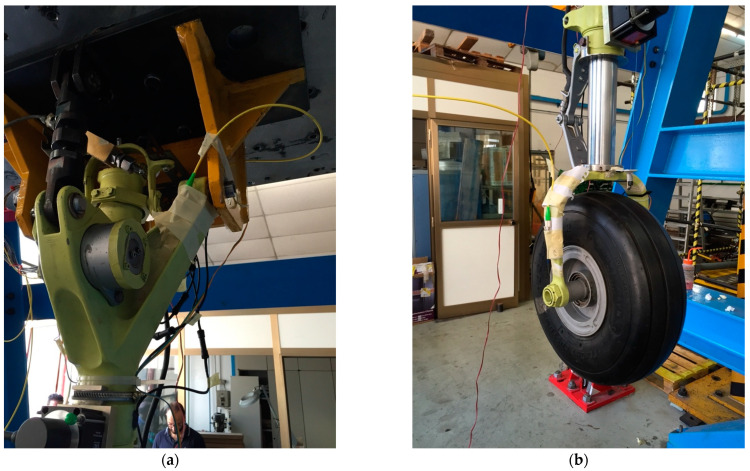
Details of the FBG sensors installed on the body (**a**) and on the fork (**b**) of the landing gear during the drop tests.

Drop test were carried out by a modified Charpy impact system. By counter rotating the pendulum hammer, it is possible to install a cylindrical block having different sizes and shapes, usually a hemispherical head. The component was located on the vertical surfaces of the supporting bases of the pendulum at the height of the notched plane of the hammer (0° angle). The electromagnetic brake limits the phenomenon of bouncing. The drop masses have different geometries and weights according to the different energy levels and damage required for the test. The test setup is shown in Figure 22, where the presence of the FBG sensors is highlighted.

Due to the generation of a sinusoidal (harmonic) loading in the range of 5–500 Hz, a vibration test was performed in such a way that the component experienced a resonance state. The mentioned frequency range corresponds to the possible scenario in which the component would be undergone.

**Figure 23:**
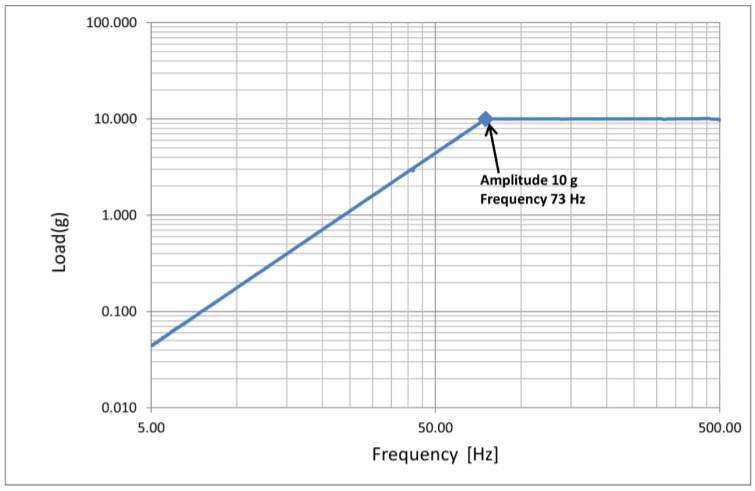
Acceleration spectrum during the vibration tests.

Figure 23 shows the acceleration spectrum applied to the composite drag brace during the vibration test. It is worth saying that the component under test was first tested as manufactured and, for a second time, after an impact event to underline the differences in its dynamic behavior.

**Figure 24:**
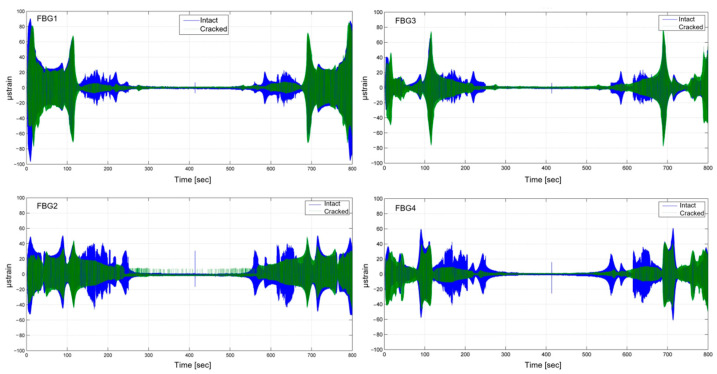
Strain history during sine sweep test.

As reported in Figure 24, four FBG sensors were installed and bonded on the component. In section AA’, two FBG sensors acting as deformation sensors were integrated for the side. In Figure 24, the strain measured on the whole component is shown. A little discrepancy in the deformation intensity can be noted during the test: sensors on the front side show a lower deformation.

In this work, a composite component has been studied as the retrofit of a metallic brag for the civil aircraft industry. FBG sensors have been employed for the identification of damaged status. With sinusoidal and random vibrations, the dynamic behavior has been investigated. To induce damage in the component, a modified Charpy hammer has been used. The results show that the impact induced a permanent strain within the component with a slight negative permanent deformation on the impacted side and a positive residual strain on the opposite one.

Since the damaged structure affects the component’s natural frequencies, it is possible to identify it through a frequency response: a clear modification of the spectrum is present between 300 and 500 Hz. Moreover, after the impact event, a frequency shift of 30 Hz in the impacted component behavior was revealed. The latter can be identified through the FBG sensors installed.

Finally, this methodology has been proved to be a simple and alternative procedure, through the help of an FBG-based sensor network, for the modal characteristics determination of a composite component.

## 3. Marine Applications: Fiber-Optic Hydrophones

A hydrophone is a sensor devoted to listening to underwater sound for civil or military applications. Most hydrophones are based on piezoelectric transducers (commonly abbreviated as PZT) that generate an electric potential when subjected to a pressure change. Piezoelectric hydrophones currently have excellent performance but still have some limitations [95]. PZT hydrophones are sensitive to electromagnetic interference, require complex data acquisition systems, have poor multiplexing capabilities, and are unsuitable for harsh environments [96,97].

Fiber-optic sensor technology represents a valid alternative solution to conventional systems for underwater applications. The use of optical fiber allows the overcoming of important limitations. In fact, fiber-optic hydrophones are light, not bulky, and they have several potential advantages, such as high sensitivity, large dynamic range, small size, lightweight, and immunity to electromagnetic interference. Bucaro et al. were the first to propose the idea of employing optical fiber technology for developing hydrophones [98]. The scientific literature reports countless studies carried out on fiber-optic hydrophones [99]. Many research groups have proposed different configurations of fiber-optic sensors to develop hydrophones for underwater applications. A recent review from Meng et al. [99] provided a survey on fiber-optic hydrophones. Here, we report for brevity only the main sensing typologies.

Hydrophones based on distributed feedback fiber laser (DFB FL) as sensing elements demonstrated high performances and multiplexing capability. Hill et al. [100], indeed, first reported a DFB FL hydrophone extremely sensitive to acoustic perturbations. In the following years, a DFB FL sensor was developed by Foster et al. [101], and an in-field demonstration of an FL hydrophone array was reported [102] too.

More simple configurations were proposed exploiting FBGs as sensing elements. Takahashi et al. developed an optical fiber hydrophone with FBGs [103,104,105,106]. In order to enhance the hydrophone response, Takashi et al. [107] used a time-division-multiplexing operation of temperature compensation with feedback control. Following this approach, Cranch et al. [108] and then Dong et al. [109] validated time-division and wavelength-division strategies for multipoint sensing, using FBGs as sensing elements for hydrophone devices. Nonetheless, the elastic modulus of the material of which the FBGs are made, i.e., glass, is high. This feature limits the performance of FBGs such as a hydrostatic pressure sensor in underwater applications. Therefore, they proposed to cover FBGs with a material with a lower Young’s modulus than glass to provide a greater sensitivity to hydrostatic pressure. Several years later, Moccia et al. carried out a systematic study to identify an optimum coating suitable to improve and tailor the FBG response to an underwater acoustic wave [110]. Based on such studies, optical fiber hydrophones FBGs coated with compliant overlay were developed and tested in the sea, obtaining a resolution of 10 mPa/√Hz [111].

Hydrophones exploiting interferometric schemes were proposed too. To date, Digonnet et al. developed a Fabry–Perot interferometric hydrophone featuring a compliant mirror (i.e., a photonic-crystal diaphragm) suspended on a single-mode optical fiber end facet, demonstrating an average minimum detectable pressure of 90 μPa/√Hz in the frequency range 100 Hz–2.5 kHz [112,113,114,115].

Furthermore, re-proposing the original idea of Bucaro, Nash et al., realized an interferometric wound-coil hydrophone operating at a sea state zero (SSZ) resolution in the frequency range 20 Hz–1 kHz [116,117]. In-field demonstration of an array in the sea close to the coast was reported too [118]. A seismic fiber-optic hydrophone was also developed and tested in the sea for volcanological monitoring applications [119].

### 3.1. Fiber-Optic Hydrophones for Towed Array Applications

Recently, the Optoelectronic Division—Engineering Department of University of Sannio, Benevento, developed fiber-optic hydrophones (FOH) based on an interferometric fiber coil around a composite mandrel. The proposed sensing configuration was exploited also to develop a linear array configuration for towed applications. Indeed, the growing demand of unmanned underwater vehicles with small size dimensions emphasized the need for compact and light towed arrays of hydrophones. The characterization of the developed fiber-optic hydrophones in terms of responsivity, linearity, and directivity has been reported. Experimental tests with FOH were conducted in an instrumented tank at Leonardo Spa—Italy [120].

### 3.2. Interferometric Fiber-Optic Hydrophone

**Figure 25:**
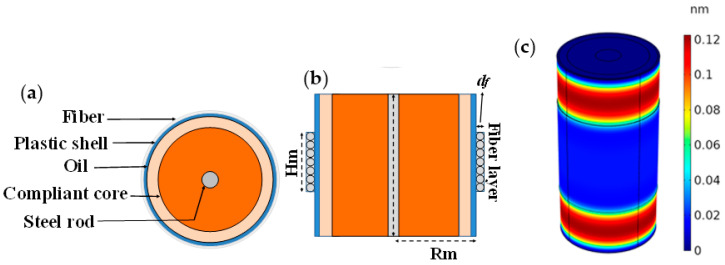
(**a**) Transversal and (**b**) lateral side view of the composite mandrel hydrophone. (**c**) 3D radial displacement pertaining to the FOH under 1 Pa static pressure. Reproduced with permission from ref. [120] (the figure is released under a Copyright Clearance Center’s RightsLink^®^ service).

The FOH has a cylindrical shape, and it is constituted of a composite mandrel. In Figure 25a,b, we display the schematization of the FOH. The structure of the hydrophone relies on a plastic shell filled with oil and a compliant solid core. A steel rod at the center serves to support the structure. Around the plastic shell, an optical fiber is suitably wounded.

An acoustic wave with a wavelength larger than the hydrophone size behaves like a hydrostatic pressure for the hydrophone, that is, a uniform force externally exerted on the FOH surface, such as to deform the mandrel. The deformation involves either an expansion or a compression of the mandrel, which results in a strain in the optical fiber around it [120].

We used two FBGs at different wavelengths to spectrally mark the extremities of the optical fiber coil. A Michelson interferometric scheme is used to retrieve the fractional length changes of the marked optical fiber versus time. A “dummy” hydrophone is used as a reference arm.

A full 3D numerical analysis was preliminarily performed by finite element methods in order to properly select the physical and geometrical properties. Specifically, the hydrophone design was devoted to obtaining a sensitivity as high to possess self-noise levels below sea-state zero and to offer a robust package for underwater operation. The final sensing configuration features a fiber coil covering the plastic shell for a height of 20 mm and a mandrel radius of 10 mm. In Figure 25c, we report the radial displacement of the hydrophone under 1 Pa static pressure. The corresponding optical fiber length is 12 m. The radial displacement is about 0.1 nm/Pa and is nominally suitable to attain a responsivity of 23 nm/Pa and the desired resolution.

### 3.3. Field Trial Validation

**Figure 26:**
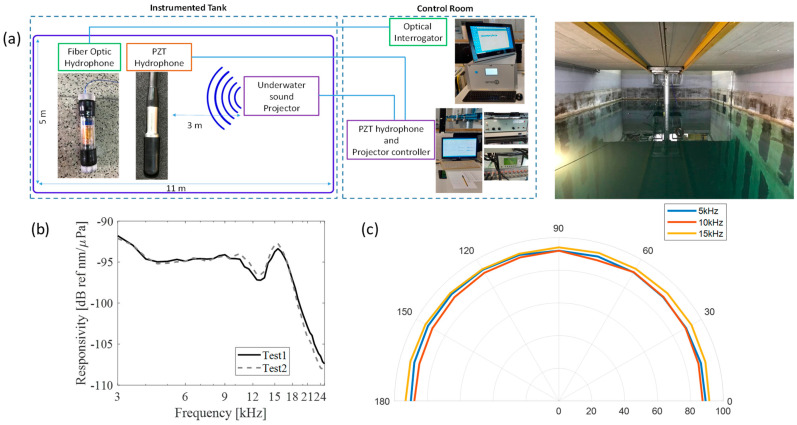
(**a**) Schematic of the experimental setup. The instrumented water tank is large 11 m × 5 m with a depth of 7 m. (**b**) Experimental Responsivity in dB of FOH hydrophone in a range from 3 kHz to 25 kHz in two different characterization tests. (**c**) Directivity of the FOH at 5 kHz, 10 kHz, and 15 kHz. Reproduced with permission from ref. [120] (the figure is released under a Copyright Clearance Center’s RightsLink^®^ service).

In order to assess the performances of the realized FOHs, the fabricated FOHs were characterized at the Leonardo Spa. In Figure 26, we show the schematic representation of the setup and the instrumented tank used for the characterizations. The tank, filled with saline water, is equipped with an acoustic source and a reference PZT hydrophone. The FOH and the PZT hydrophone are positioned in close proximity at a distance of 3 m from the acoustic sound projector, and all three devices are at a depth of 3 m. The optical interrogator [120], the PZT hydrophone controller, and the source controller are positioned in a control room. The underwater acoustic source was set to radiate short sinusoidal acoustic pulses in the frequency range 3–20 kHz with 500 Hz steps. A PZT (responsivity: −205 dB re 1 V/µPa—voltage gain: 50 dB) was used as a reference hydrophone.

The responses of PZT and FOH were recorded, and the responsivity was experimentally retrieved (as impulse amplitude measured by the optical hydrophone divided for the impulse amplitude measured by the reference PZT hydrophone). In Figure 26b, we display the resulting FOH responsivity in [nm/Pa]. We can see that the FOH is characterized by a responsivity of about 19 nm/Pa in the frequency range 3 ÷ 10 kHz, allowing a resolution of 0.3 mPa/Hz^1/2^. We also verified the FOH sensors linearity. Additionally, in order to assure the omnidirectional response of the FOH, a motorized stage was used to rotate the FOH with respect to the source. The directivity of the FOH at 5 kHz, 10 kHz, and 15 kHz, is shown in Figure 26c.

**Figure 27:**
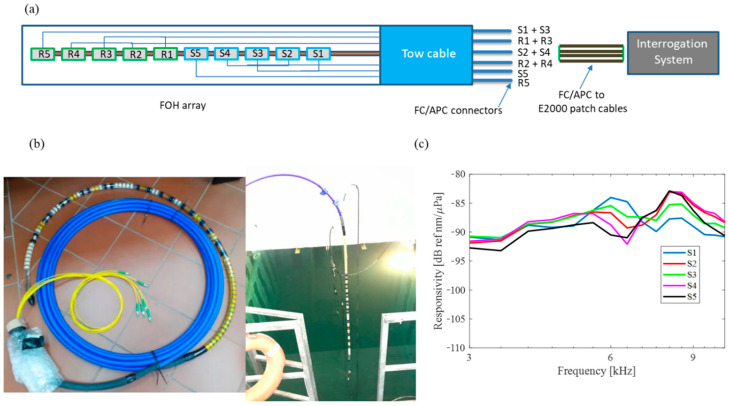
(**a**) Schematization of FOH in array configuration (sensitive hydrophones are labeled with “S”, reference hydrophones with “R”), (**b**) FOH array, and (**c**) Experimental responsivity in dB of FOH array. Reproduced with permission from ref. [120] (the figure is released under a Copyright Clearance Center’s RightsLink^®^ service).

Finally, a five-element towed array was fabricated using the FOH as the basic building block. The array topology is shown in Figure 27a. The tow-cable has six optical fibers running through its entire length. On one side, these fibers are spliced to the hydrophones in the array. On the other end, the fibers are spliced to six simplex optical fibers with FC/APC termination. A photo of our developed FOH array is reported in Figure 27b.

The hydrophones multiplexed in the towed array were simultaneously interrogated by using the same interrogation interferometric readout system and exploiting its multiplexing capabilities. Following the same procedure adopted for a single FOH, the array elements were characterized, and their responsivities were reported in Figure 27c, confirming the previous performances and witnessing good process repeatability.

In brief, we have reported the results of a performance analysis of FOHs, carried out within the frequency range of 3 ÷ 25 kHz at Leonardo Finmeccanica premises. The FOH exhibited a responsivity of about 19 nm/Pa with a resolution of 0.3 mPa/Hz^1/2^ at the SSZ level. The experimental results in the field, in terms of responsiveness, linearity, and directivity, obtained for a single FOH, have led to further experimental tests for the characterization of a FOH array. Preliminary results of a FOH array tested using an instrumented tank provided further confirmation of the great potential of the proposed technology for practical acoustic monitoring applications.

## 4. Conclusions and Future Trends

The employability of photonics technology in today’s era has been gaining increasing importance in aerospace and submarines. They have been an appealing challenge for the scientific communities. On this line of argument, in this paper, we have reviewed our main results achieved so far on the use of optical fiber sensors for safety and security in innovative aerospace and submarine applications. Other related applications have been reported in the companion papers [49,50] published in this Special Issue.

Some of our results have been exploited in new market devices, thus cooperating in the creation of some start-up companies (OFTEN MEDICA, OPTOSMART, OPTOSENSING, BIOTAG), working in medical applications and in the design and application of point-based and distributed optical fiber sensors, as well as by the construction of the Center of Nanophotonics and Optoelectronics for Human Health and Industrial Applications (CNOS) which will be completed by April 2023 [49].

The main goals of our future activity can be divided along three main lines:

1. To translate the innovative research results into other market products, thus creating new start-up companies;
2. To explore new applications to improve both the safety and the security in other fields, such as agrifood, antiterrorism, biomedical devices, precision medicine, environment, and energy saving;
3. To improve the performance of our devices by increasing the use of both the nanotechnology and the nanomaterials.

## Data Availability

No new data were created or analyzed in this study. Data sharing is not applicable to this article.
